# Correlation between MGMT methylation and the efficacy of bevacizumab in high-grade glioma

**DOI:** 10.3389/fonc.2025.1644934

**Published:** 2025-09-02

**Authors:** Chengrui Yan, Xuexue Bai, Chao Wu, Wenbin Ma, Ming Feng

**Affiliations:** ^1^ Department of Neurosurgery, Peking Union Medical College Hospital, Chinese Academy of Medical Science and Peking Union Medical College, Beijing, China; ^2^ Department of Neurosurgery, Tengzhou Central People’s Hospital, Tengzhou, Shandong, China

**Keywords:** bevacizumab, high-grade glioma, temozolomide, methylation, predictor

## Abstract

**Purpose:**

The aim of this study is to investigate whether the therapeutic efficacy of bevacizumab (BEV) in the treatment of high-grade glioma (HGG) is associated with the methylation status of O6-methylguanine-DNA methyltransferase (MGMT) on the basis of excluding the interference from chemotherapy drugs.

**Methods:**

This study included 57 patients with histologically confirmed HGG who, due to various reasons, were unable to complete the standard chemotherapy protocol and thus received BEV treatment. Patients enrolled in the study were divided into two groups based on their MGMT status: the unmethylated MGMT group and the methylated MGMT group. Depending on whether the numerical variables of the patients satisfied a normal distribution, either the T-test or the rank-sum test was employed. For the comparison of categorical variables, the chi-square test was used.

**Results:**

After BEV treatment, both PFS and OS were higher in the methylated MGMT group compared to the unmethylated group. Additionally, the tumor control rate was also higher in the methylated group. Furthermore, in both patient groups, a decrease in steroid dosage was observed following BEV treatment, accompanied by an increase in KPS. Multivariate COX regression analysis revealed that radiotherapy and complete tumor resection were significant factors influencing PFS and OS in HGG patients. Additionally, pathological grade was found to influence PFS in HGG patients.

**Conclusion:**

Based on the exclusion of the interference from chemotherapy drugs, our study is the first to confirm the correlation between the methylation status of MGMT and the therapeutic effect of BEV on HGG.

## 1 Introduction

High-grade gliomas (HGG) are a common intracranial malignancy. The standard treatment typically involves surgical resection followed by synchronous radiotherapy and chemotherapy, with continued adjuvant chemotherapy thereafter (1–3). Temozolomide (TMZ) remains the most frequently employed chemotherapeutic agent in the management of these aggressive tumors (4, 5). A pivotal factor influencing the therapeutic response to TMZ is the methylation status of the O6-methylguanine-DNA methyltransferase (MGMT) gene (6). However, TMZ use is frequently associated with treatment discontinuation due to thrombocytopenia and other adverse effects (7). Bevacizumab (BEV), a monoclonal antibody targeting vascular endothelial growth factor (VEGF), has emerged as a promising agent in the treatment paradigm of HGG, demonstrating potential in prolonging progression-free survival, albeit with equivocal impacts on overall survival (8–10). From a biological perspective, MGMT promoter methylation leading to gene silencing is not only associated with alkylating agent resistance but may also influence anti-angiogenic therapy by altering tumor angiogenesis characteristics (11). Studies suggest that MGMT methylation status may be potentially linked to aberrant activation of the VEGF pathway in the tumor microenvironment, with methylated tumors potentially exhibiting greater dependence on angiogenesis, thereby increasing sensitivity to VEGF inhibitors like BEV (12). Previous studies involving the relationship between MGMT methylation and the efficacy of BEV have been confounded by various chemotherapy agents. Consequently, the correlation between BEV’s therapeutic effect and MGMT methylation remains elusive. In this retrospective study, we aimed to investigate whether the methylation status of MGMT influences the therapeutic response of HGG to BEV, after excluding the interference of chemotherapy drugs. Our ultimate goal is to inform personalized treatment strategies for these patients.

## 2 Materials and methods

### 2.1 Patients

**Figure 1:**
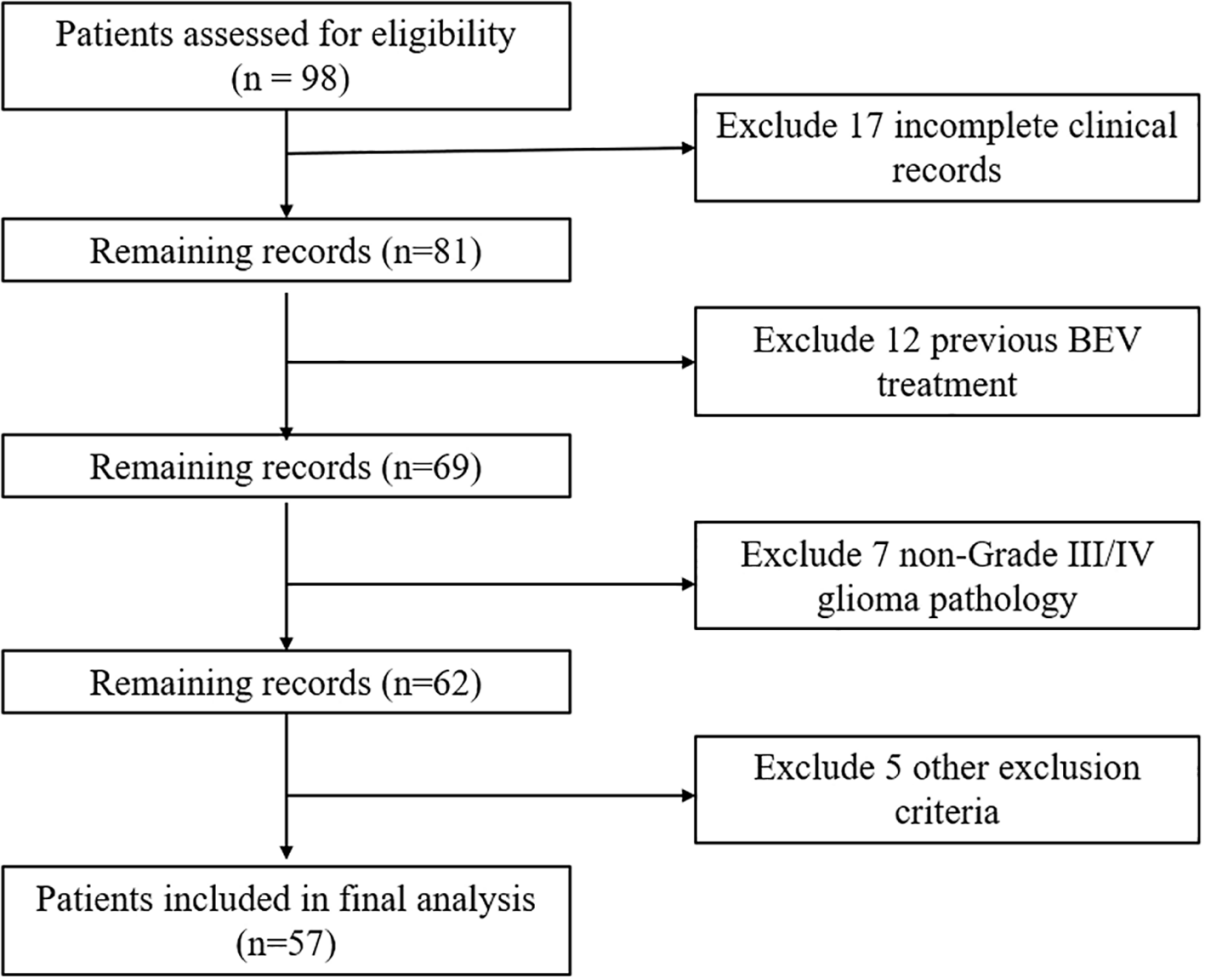
CONSORT flow diagram of patient screening and enrollment.

Inclusion Criteria: (1) Patients must have a confirmed pathological diagnosis of grade III or IV glioma. (2) Only adult patients aged 18 years or older will be included. (3) Patients must have received standard treatment for glioma but must not have undergone treatment with BEV. (4) Patients must possess comprehensive clinical data. Exclusion Criteria: (1) Patients with a recent history of abnormal bleeding will be excluded due to potential contraindications with study treatments. (2) Patients with concurrent malignancies other than the primary glioma diagnosis will be excluded. (3) The presence of severe systemic diseases that could significantly impact patient outcomes or interfere with the study assessments will result in exclusion from the study. **Figure 1** shows the CONSORT flow diagram of patient screening and enrollment. A total of 57 patients were included in this study. All these patients had received surgery but started to receive BEV treatment after stopping TMZ treatment due to various reasons. **Table 1** summarizes the detailed demographic characteristics of the enrolled patients. This study adhered to the principles of the Declaration of Helsinki and followed the guidelines of the Strengthening the Reporting of Observational Studies in Epidemiology (STROBE). All data utilized in this study were de-identified to ensure the privacy and confidentiality of human subjects. The study protocol was reviewed and approved by the Ethics Committee of Peking Union Medical College Hospital, Chinese Academy of Medical Sciences (Approval No. ZQ-3194). Written informed consent was obtained from all participants and/or their legal guardian(s) prior to the study.

**Table 1:** Baseline demographic characteristics of all patients.

Parameter	MGMT		P-value
	Unmethylation (n=26)	Methylation (n=31)	
Age (mean, years)	48.50±12.44	52.68±10.02	0.178
Sex (N)			0.749
Male	12	13	
Female	14	18	
Pathological grade (N)			0.903
WHO III	13	15	
WHO IV	13	16	
Extent of resection (N)			0.949
Complete resection	21	24	
Subtotal resection	3	4	
Partial resection	2	3	
IDH (N)			0.860
Mutation	7	9	
Wild type	19	22	
Tumor volume (ccm)	29.92±12.24	27.32±10.33	0.574
Radiotherapy (N)	22	23	0.336
KPS	63.46±14.13	64.19±13.61	0.860
TMZ cycle	1.42±0.73	1.21±0.77	0.211
TMZ Cessation Reason			0.821
Hematologic toxicity	16	18	
Gastrointestinal events	6	8	
TMZ resistance	4	5	
BEV cycles	5.13±2.14	4.96±1.97	0.421
Steroids (mg)	36.15±8.04	33.55±9.50	0.270

MGMT, O6-methylguanine-DNA methyltransferase; WHO, world health organization; IDH, isocitrate dehydrogenase; KPS, karnofsky performance score; TMZ, temozolomide.

### 2.2 Therapeutic process

All enrolled HGG patients underwent surgical resection followed by TMZ chemotherapy. Of these, 45 patients received postoperative radiotherapy. All participants completed the standard 5-day TMZ regimen (28-day cycles) (13). The maximum number of cycles for TMZ treatment in this study was 3. Due to hematologic toxicity, drug resistance, and other reasons, these patients discontinued TMZ treatment after receiving various cycles of the drug and began BEV therapy. The recommended BEV dosage in previous studies was 5-15 mg/kg (14–16). In this study, all patients received a BEV dosage of 5 mg/kg, administered every two weeks.

### 2.3 Efficacy evaluation

Imaging studies were completed within three days post-surgery, utilizing t1-weighted enhancement sequences to assess tumor status (17). Complete resection was defined as postoperative absence of tumor enhancement on imaging. Subtotal resection indicated residual enhancement ≤5% of preoperative tumor volume, while partial resection represented residual enhancement >5%. Follow-up imaging was performed at 1- to 2-month intervals (18). Therapeutic response was evaluated according to the Response Assessment in Neuro-Oncology (RANO) criteria (19). Complete response (CR) was defined as the disappearance of tumor signals, partial response (PR) as a reduction in tumor area by at least 50%, stable disease (SD) as a reduction in tumor size by less than 50% or an increase by less than 25%, and disease progression (PD) as an increase in tumor size by at least 25% (20). The overall response rate (OR) encompassed both CR and PR. Progression-free survival (PFS) and overall survival (OS) were calculated from the beginning of BEV treatment.

### 2.4 Statistical analysis

Continuous variables were expressed as mean ± standard deviation (mean ± SD). All categorical variables were described using the number of patients or percentages (%). The data of all continuous variables were first tested for normal distribution. If the data followed a normal distribution, the t-test was used for comparison. If the data did not conform to a normal distribution, the rank-sum test was employed for comparison. The chi-square test was used for the comparison of categorical variables. Univariate and multifactorial COX regression analyses were used to explore independent risk factors for PFS and OS in HGG patients. The survival curves for PFS and OS were plotted using the Kaplan-Meier method. All data in this study were analyzed using SPSS (version 27.0, IBM). A P value < 0.05 was considered statistically significant.

## 3 Results

### 3.1 Baseline characteristics

All patients received TMZ therapy postoperatively, and 45 patients also underwent radiotherapy. During the course of treatment, all patients stopped receiving TMZ treatment and began BEV treatment due to severe adverse reactions such as hematologic toxicity, vomiting, and other reasons. Prior to BEV treatment, the number of TMZ cycles in the MGMT unmethylated group and the methylated group were 1.42 ± 0.73 and 1.21 ± 0.77 cycles, respectively (P > 0.05). The mean number of BEV cycles administered was comparable between groups, with 4.96 ± 1.97 cycles in the methylated cohort versus 5.13 ± 2.14 cycles in the unmethylated cohort (P > 0.05), confirming equivalent treatment exposure. Among the 26 patients in the MGMT unmethylated group, 2 (7.7%) experienced tumor recurrence. In the 31 patients of the methylated group, 3 (9.7%) had tumor recurrence. The results showed no significant differences in baseline characteristics between the two groups prior to BEV treatment.

### 3.2 Cox regression analysis

This study employed COX regression analysis to investigate the risk factors for PFS and OS in patients with HGG. Univariate analysis revealed that high pathological grade and large tumor volume were risk factors for both PFS and OS, while radiotherapy, complete tumor resection, and preoperative KPS score > 70 were protective factors for PFS and OS in patients with HGG. Multivariate analysis further indicated that pathological grade (HR 2.41, 95% CI 1.29-4.48, p=0.006), radiotherapy (HR 0.34, 95% CI 0.15-0.79, p=0.011), and complete resection (HR 0.32, 95% CI 0.14-0.73, p=0.007) were significant predictor of PFS. Additionally, multivariate analysis demonstrated that radiotherapy (HR 0.45, 95% CI 0.20-0.99, p=0.048) and complete resection (HR 0.32, 95% CI 0.14-0.73, p=0.007) were significant predictor of OS. **Table 2** summarizes the results of both univariate and multivariate COX regression analyses for PFS. **Table 3** presents the results of both univariate and multivariate COX regression analysis for OS.

**Table 2:** Univariate and multivariate cox regression analysis of progression-free survival.

Variables	N	Univariate		Multivariate	
		HR (95%CI)	p Value	(95%CI)	p Value
Age	57	1.00 (0.98; 1.03)	0.747		
Gender	57	1.25 (0.72; 2.18)	0.436		
Pathological grade	57	2.23 (1.27; 3.92)	0.005	2.41 (1.29; 4.48)	0.006
Radiotherapy	45	0.21 (0.10; 0.44)	0.001	0.34 (0.15; 0.79)	0.011
Complete resection	37	0.30 (0.15; 0.59)	0.001	0.32 (0.14; 0.73)	0.007
KPS>70	36	0.40 (0.22; 0.73)	0.003	0.58 (0.29; 1.14)	0.114
Tumor>30ccm	20	2.22 (1.21; 4.05)	0.010	1.80 (0.92; 3.52)	0.087

These variables with a P-value<0.05 in the univariate analysis were included in the multivariate Cox regression analysis.

HR, hazard ratio; CI, confidence interval; KPS, karnofsky performance score.

**Table 3:** Univariate and multivariate cox regression analysis of overall survival.

Variables	N	Univariate		Multivariate	
		HR (95%CI)	p Value	(95%CI)	p Value
Age	57	1.01 (0.98; 1.03)	0.683		
Gender	57	1.26 (0.71; 2.24)	0.435		
Pathological grade	57	1.80 (1.01; 3.21)	0.048	1.66 (0.88; 3.14)	0.119
Radiotherapy	45	0.28 (0.14; 0.58)	0.001	0.45 (0.20; 0.99)	0.048
Complete resection	37	0.27 (0.13; 0.54)	0.001	0.32 (0.14; 0.73)	0.007
KPS>70	36	0.43 (0.24; 0.77)	0.005	0.63 (0.32; 1.25)	0.187
Tumor>30ccm	20	2.17 (1.18; 3.98)	0.013	1.38 (0.69; 2.77)	0.364

These variables with a P-value<0.05 in the univariate analysis were included in the multivariate Cox regression analysis.

HR, hazard ratio; CI, confidence interval; KPS, karnofsky performance score.

### 3.3 Therapeutic effect

**Figure 2:**
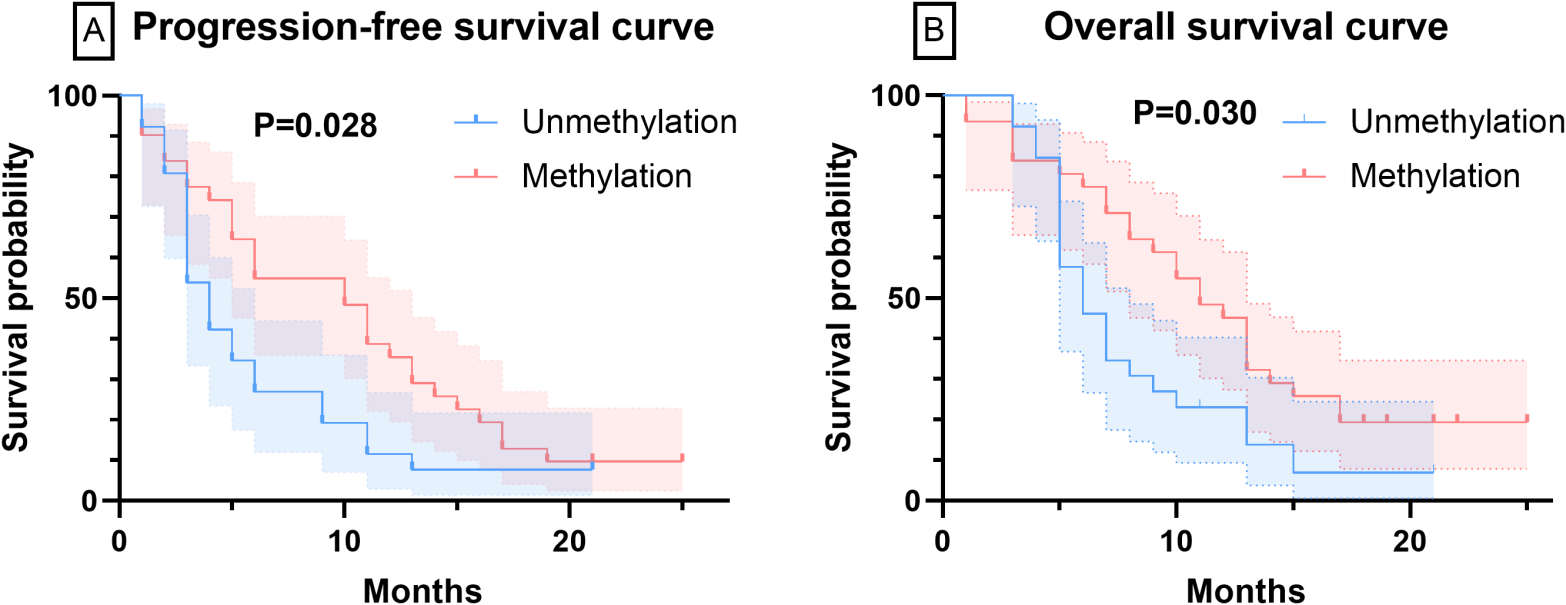
The curves of PFS and OS were plotted by Kaplan-Meier method. **(A)** The PFS curves of patients with varying MGMT methylation statuses. **(B)** The OS curves of patients with varying MGMT methylation statuses.

After initiating BEV treatment, the PFS for the unmethylated and methylated MGMT groups were 5.77 ± 4.75 months and 9.81 ± 6.67 months, respectively, while the OS were 7.73 ± 4.31 months and 11.42 ± 6.36 months, respectively (P<0.05). At 6 months of BEV treatment, 19 patients in the MGMT unmethylated group and 14 patients in the methylated group experienced tumor progression, with PFS of 26.9% and 54.8%, respectively (P<0.05). **Figure 2** displays the PFS and OS curves for patients with different MGMT statuses. Regarding the control rates, after 6 months of BEV treatment, 26.9% and 7.7% of patients in the MGMT unmethylated group achieved CR and PR, respectively. Twelve patients achieved SD, while the remaining patients exhibited PD. In the MGMT methylated group, 54.8% and 19.4% of patients achieved CR and PR, respectively. Five patients exhibited SD, and the remaining three patients exhibited PD. In summary, the OR for the MGMT unmethylated group was 34.6%, while the OR for the methylated group was 74.2% (P<0.05).

### 3.4 Clinical symptoms

Prior to BEV treatment, all patients received steroidal hormones (methylprednisolone) to manage clinical symptoms. Specifically, patients in the MGMT unmethylated group received a steroidal dose of 36.15 ± 8.04 mg, while those in the methylated group received 33.55 ± 9.50 mg. After BEV treatment, the steroidal dose decreased significantly in both groups, with the unmethylated group receiving 21.54 ± 13.77 mg and the methylated group receiving 18.71 ± 15.44 mg (P > 0.05). Prior to BEV treatment, the KPS score in the unmethylated group was 63.46 ± 14.13, while that in the methylated group was 64.19 ± 13.61. Following BEV treatment, the KPS scores increased in both groups, with the unmethylated group scoring 71.15 ± 8.64 and the methylated group scoring 73.23 ± 13.51 (P > 0.05).

### 3.5 Adverse drug reactions

BEV-associated adverse effects included various types of bleeding, headaches, hypertension, blood toxicity, thrombosis, proteinuria, gastrointestinal perforation, delayed wound healing, congestive heart failure, sepsis, and nephrotic syndrome (21, 22). In this study, hypertension was the most common adverse reaction, occurring in 11 patients. Additionally, 5 patients exhibited headache, 2 patients developed hematological toxicity, and 1 patient presented with proteinuria. No adverse reactions occurred that were severe enough to necessitate discontinuation of BEV treatment.

## 4 Discussion

After receiving BEV treatment, patients in both groups exhibited a significant decrease in steroid dosage. Concurrently, there was varying degrees of improvement in clinical symptoms, with the majority of patients maintaining a high KPS level similar to ​the​ level before treatment or even achieving further improvements. Previous studies have also demonstrated that BEV can effectively alleviate peritumoral edema in patients with HGG, thereby improving their clinical symptoms (23–25). No significant differences were observed between the two groups in terms of steroid dosage and KPS. To explore the factors influencing PFS and OS in HGG patients, we employed COX regression analysis. Univariate analysis revealed that pathological grade, tumor volume, radiotherapy, complete tumor resection, and KPS were significant factors affecting PFS and OS in HGG patients. Multivariate analysis further indicated that radiotherapy and complete tumor resection were the main influencing factors for both PFS and OS. These findings are consistent with previous studies (26). Additionally, pathological grade emerged as a factor influencing PFS but not OS.

Analysis of our cohort demonstrated markedly reduced progression-free and overall survival durations in WHO CNS grade 4 glioma patients relative to grade 3 cases, consistent with established prognostic correlations in current neuro-oncology practice (27). The clinical relevance of histopathological grading emerges through multiple mechanisms: foremost, advanced-grade neoplasms demonstrate enhanced microvascular proliferation and necrosis patterns- morphological hallmarks of tumor aggressiveness (28, 29). Additionally, progressive histological malignancy shows strong associations with amplified cellular proliferation indices and chromosomal instability. Importantly, our adjusted regression models established pathological grading as maintaining independent predictive value for disease progression even when accounting for MGMT epigenetic status.

Standard treatment for HGG typically involves maximal surgical resection followed by six weeks of concurrent radiotherapy and TMZ, followed by six months of adjuvant TMZ chemotherapy (30). It is well-established that MGMT methylation is a crucial prognostic predictor for HGG patients receiving TMZ treatment (31). However, in our study, the patients received a much lower dose of TMZ than the standard TMZ protocol, therefore, we believe that the interference of TMZ on the study results was limited. In this study, a single-center retrospective analysis was conducted on 57 patients with HGG who underwent BEV treatment. The results indicated that the PFS of the non-methylated group was 5.77 months, whereas the PFS of the methylated group was 9.81 months. Additionally, the OS of the non-methylated and methylated groups were 7.73 months and 11.4 months, respectively. Significant differences in PFS and OS were observed between the two groups, suggesting that HGG patients with MGMT methylation respond better to BEV treatment than those without methylation. Furthermore, notable disparities were also observed in tumor control rates between the two groups. Specifically, the OR of the unmethylated MGMT group was 34.6%, while the OR of the methylated group was 74.2%. Therefore, we propose that MGMT methylation serves as a predictor for the therapeutic response of BEV in patients with HGG.

In a study involving 191 patients, the authors analyzed the predictive value of MGMT status on tumor prognosis in patients with recurrent HGG treated with BEV (32). Their research demonstrated that MGMT status was not associated with patient prognosis. Although the number of cases was substantial, it is noteworthy that the MGMT status of 63% of the patients in their study was unknown. A separate multicenter retrospective study evaluated the effectiveness of BEV in treating patients with recurrent glioblastoma. In this study, the rates of IDH and MGMT status were 74% and 83%, respectively. However, the authors did not analyze the predictive effect of MGMT status on prognosis (33). In a study involving 437 patients with recurrent glioblastoma, the authors demonstrated that the progression-free survival (PFS) was prolonged with the combination of lomustine and BEV compared to lomustine alone. However, no survival advantage was observed. Notably, in this study, patients with MGMT-methylated recurrent glioblastoma had a PFS twice as high as those without methylation (34). Nevertheless, it remains uncertain whether this difference was exaggerated by the effect of lomustine. In another study encompassing 92 patients with recurrent HGG, the authors reported a higher incidence of MGMT methylation among long-term responders (PFS ≥ 6 months) (35). However, it is worth mentioning that some patients in this study received concurrent treatment with TMZ or other chemotherapy agents such as fotemustine. The prognostic and predictive role of TMZ in HGG with MGMT methylation is well-established (36, 37). Therefore, we believe that the results of this study may have been influenced by the chemotherapy agents.

In our study, all patients discontinued treatment with TMZ at far lower doses than the standard protocol due to various reasons. Consequently, the impact of chemotherapy agents on PFS and OS in HGG patients was minimal in our study. To the best of our knowledge, our study is the first to investigate the correlation between MGMT methylation and the prognosis of HGG patients treated with BEV, while excluding the interference of chemotherapy drugs. Our findings suggest that BEV is more effective in patients with MGMT-methylated HGG compared to those without methylation, indicating that MGMT can serve as a prognostic predictor for BEV treatment in HGG. However, it is important to acknowledge some limitations of this study. Firstly, as a single-center study, the results may not be fully representative. Furthermore, due to the limited number of patients who discontinued TMZ treatment, the case numbers in this study are restricted. Future research will aim to include a larger sample size to obtain more accurate results. Secondly, due to its retrospective nature, our study had limitations in evidence collection. Therefore, larger, multicenter, prospective studies with a greater sample size are needed to comprehensively evaluate the efficacy of BEV.

## 5 Conclusion

Based on the exclusion of the interference from chemotherapy drugs, our study is the first to confirm the correlation between the methylation status of MGMT and the therapeutic effect of BEV on HGG. Our results indicate that BEV is more effective in patients with MGMT-methylated HGG compared to unmethylation, suggesting that MGMT can serve as a prognostic predictor for BEV treatment in HGG.

## Data Availability

The data presented in the study are deposited in the Zenodo repository, accession number 10.5281/zenodo.16903955 (URL: https://zenodo.org/records/16903955).
